# Complete Genome Sequence of *Salmonella enterica* Serovar Typhimurium Strain SO3 (Sequence Type 302) Isolated from a Baby with Meningitis in Mexico

**DOI:** 10.1128/genomeA.00285-16

**Published:** 2016-04-21

**Authors:** Pablo Vinuesa, José L. Puente, Edmundo Calva, Mussaret B. Zaidi, Claudia Silva

**Affiliations:** a Programa de Ingeniería Genómica, Centro de Ciencias Genómicas, Universidad Nacional Autónoma de México, Cuernavaca, Morelos, Mexico; b Departamento de Microbiología Molecular, Instituto de Biotecnología, Universidad Nacional Autónoma de México, Cuernavaca, Morelos, Mexico; c Microbiology Research Laboratory, Hospital General O’Horan and Infectious Diseases Research Unit, Hospital Regional de Alta Especialidad de la Península de Yucatán, Mérida, Yucatán, Mexico

## Abstract

The complete genome of *Salmonella enterica* serovar Typhimurium strain SO3 (sequence type 302), isolated from a fatal meningitis infection in Mexico, was determined using PacBio technology. The chromosome hosts six complete prophages and is predicted to harbor 51 genomic islands, including 13 pathogenicity islands (SPIs). It carries the *Salmonella* virulence plasmid (pSTV).

## GENOME ANNOUNCEMENT

We report the complete genome sequence of *Salmonella enterica* subspecies *enterica* serovar Typhimurium strain SO3, which was isolated from the cerebrospinal fluid of a 1 month-old child who died of meningitis in Sonora, Mexico, in 2002. This strain was reported as part of an epidemiological surveillance program as SOHS 02-68 (1), and characterized by multilocus sequence typing as sequence type 302 (ST302) (2). This genotype was first described for human strains in Mexico (2), and later reported for African strains (http://mlst.warwick.ac.uk/mlst/dbs/Senterica). ST302 genotype is also closely related to ST313 human-invasive strains from Africa (3).

Genomic DNA was extracted by standard protocols (4) and sheared into ~10- to 20-kb fragments for PacBio library preparation and P6-C4 sequencing on one single-molecule real-time (SMRT) cell. The continuous-long-reads (CLRs) were assembled using the HGAP/Quiver protocol in SMRT Portal v2.3.0.140936.p4 (5), resulting in a two-contig assembly. These were circularized by trimming the terminal repeats with Minimus2 (6), and subjected to three consecutive rounds of CLRs remapping with the RS_Resequencing.1 module, yielding an assembly with 55× mean coverage. A final polishing step was performed by remapping quality-filtered Illumina HiSeq reads (PE 2 × 101 bp) onto the assembly using BWA (7), increasing its mean coverage to >450×. The alignment was passed to Pilon (8) to correct for indels and single nucleotide polymorphisms (SNPs). The size of the assembled genome is 5,006,803 bp, with a G+C content of 52 %, comprising a 4.9-Mb chromosome and a 93.8-kb *Salmonella* virulence plasmid (pSTV).

Gene calling and annotation was performed with a modified version of Prokka (9). A total of 4,980 genes, including 4,685 coding sequences (CDSs), and 11 pseudogenes were identified. Additionally, genes for 87 tRNAs, 22 rRNAs, and 1 tmRNA were annotated, plus 161 noncoding RNAs (ncRNAs), 3 clustered regularly interspaced short palindromic repeat (CRISPR) arrays, 5 riboswitches, and 442 signal peptides.

The annotation was manually curated, adding prophage predictions made by the PHAST server (10), and 51 genomic islands detected by IslandViewer3 (11). Among those, we identified and annotated 13 *Salmonella* pathogenicity islands (SPIs), including SPI-6, which encodes for a predicted type VI secretion system. Functional type III and type IV secretion systems were also predicted by querying the genome against effectiveDB (12). Six complete prophages and several phage remnants were located on the chromosome: ST104, Gifsy-2, ST64B, Gifsy-1, and the recently described P2-like phages ELPhiS (13) and FSL SP-004 (14). The phage repertoire and the pSTV sequence of strain SO3 were almost identical to the repertoire found in the genome of the other sequenced ST302 strain SO2 (15). A comparative genomics study of this and the other Mexican Typhimurium strains (16, 17) will be presented elsewhere.

### Nucleotide sequence accession numbers.

The complete sequences of the chromosome and the pSTV of *Salmonella* Typhimurium strain SO3 are available from GenBank under accession numbers CP014536 and CP014537, respectively.
